# Intradermal injection of low dose human regulatory T cells inhibits skin inflammation in a humanized mouse model

**DOI:** 10.1038/s41598-018-28346-5

**Published:** 2018-07-03

**Authors:** Sija Landman, Vivian L. de Oliveira, Piet E. J. van Erp, Esther Fasse, Stijn C. G. Bauland, Irma Joosten, Hans J. P. M. Koenen

**Affiliations:** 1 Radboud university medical center, department of Laboratory Medicine-Medical Immunology, Nijmegen, The Netherlands; 2 Radboud university medical center, department of Dermatology, Nijmegen, The Netherlands; 3 Bauland kliniek, Mill, The Netherlands

## Abstract

Recent regulatory T cell (Treg) based clinical trials support their therapeutic potential in transplantation and auto-inflammatory diseases. However, large numbers of Treg are needed to accomplish therapeutic efficacy. Local injection at the site of inflammation (targeted delivery) may lower the numbers needed for therapy. We evaluated if local delivery of low numbers of human Treg by intradermal injection was able to prevent skin inflammation, using the humanized mouse huPBL-SCID-huSkin allograft model. A dose of only 1 × 10^5^ freshly isolated, non expanded Treg injected intradermally in close proximity to the transplanted human skin prevented inflammation of the grafted tissue induced by 4 × 10^7^ IP injected human allogeneic PBMCs, (ratio Treg:PBMC = 1:400), as indicated by the inhibition of epidermal thickening, sustained Keratin-10 expression, the absence of Keratin-16 up regulation and prevention of human CD3+ T cell influx. A concomitant reduction of human T cells was observed in lymph nodes and spleen of the mice. Injection of Treg at the contralateral side was also shown to inhibit skin inflammation, suggesting that the inflammatory response was regulated both locally and systemically. In conclusion, local application of Treg may be an attractive way to suppress inflammation *in vivo* without the need for prior *ex vivo* expansion.

## Introduction

Regulatory T cells (Treg) are important in maintaining immune homeostasis^1^. Due to their potential as immune modulators, Treg are studied widely for therapeutic use in order to control unwanted immune responses and to promote tolerance in transplantation, autoimmunity, and chronic inflammatory diseases^2–5^.

Recently, a promising effect of Treg therapy with expanded FOXP3+ Treg in the prevention of graft-versus-host-disease, a common complication after allogeneic stem cell transplantation, was demonstrated^6,7^. Currently, Treg therapy is also tested in solid organ transplantation. In living donor liver transplantation, a pilot study with *ex vivo* expanded Treg was shown to be effective and to induce operational tolerance^8^. The ONE study investigates the use of expanded Treg after living donor kidney transplantation (clinicaltrials.gov NCT02085629). These phase I/II clinical trials aim to find the optimal dose and dosing regimen as well as to characterize the best Treg subtype and *ex vivo* expansion protocol. All these studies make use of *ex vivo* Treg expansion protocols, and struggle to consistently obtain sufficient cell numbers. Also in autoimmunity and chronic inflammatory diseases, the first trials are underway. Promising results were obtained in small scale phase I/II clinical trials in type 1 diabetes (T1DM)^9,10^, Crohn’s disease^11^ and uveitis^12^. Treg function in these diseases is often hampered and optimal isolation, expansion, and injection protocols are under study^13^. As to our knowledge, no clinical studies so far have been performed in skin inflammatory diseases, like psoriasis, while also here Treg therapy may hold promise^14^.

Currently, in clinical trials, typically around 1–10 × 10^6^ Treg/kg are infused systemically^2^. This amount of cells cannot be obtained by simple blood collection and therefore Treg have to be expanded. Different *ex vivo* expansion protocols are being studied^15–18^, however, expansion has its disadvantages. Expansion is time consuming and limits the possibilities for use in acute cases. Furthermore, due to their plasticity, Treg can convert into potentially inflammatory IL-17 producing cells, which is more likely to occur when cells are expanded^19^. When cells are given systemically/ IV, the majority of cells is lost or does not reach the site of inflammation. Therefore, we investigated the possibility of a more targeted delivery approach, with injection of Treg in or close to the site of inflammation. This approach might require lower Treg numbers compared to systemic infusion and exclude the need for expansion. To test our hypothesis we made use of a humanized mouse model for skin inflammation. The choice for skin as a model to explore local application of Treg was made on the basis that it is easy accessible and supported by the fact that Treg in skin are highly important for tissue repair and reduction of skin inflammation^20^. We used the so-called huPBL-SCID-huSkin allograft model.

In this model, human skin is transplanted to immune deficient mice and allowed to heal for 3 weeks. Thereafter, allogeneic peripheral blood mononuclear cells (PBMC) are infused by intraperitoneal (IP) injection. In time (2–3 weeks) an immune response mainly consisting of human T cells establishes as demonstrated by the influx of human T cells in the skin, peripheral blood and secondary lymphoid organs^21–23^. This human-skin allograft model resembles human plaque-type psoriasis at multiple levels, as indicated by erythema and skin thickening, acanthosis, parakeratosis and psoriasis like rete ridges, increased expression of hBD-2, K16 and reduced K10 expression and a strong influx of T cells^21^. This model also reveals similarities with the graft-versus-host-disease (GVHD) of the skin that might occur after stem cell transplantation, including increased expression of Elafin by keratinocytes^21^, which has been shown to be a biomarker of GVHD of the skin^21,24^.

In a variety of humanized mouse models, including our own, systemic injection of high numbers of Treg, i.e. Treg:PBMC ratio’s of 1:5/1:10, was required to inhibit the inflammatory response against islet^25^, arterial^26^, and skin^15,21^ allografts. Previously it has been demonstrated that following Treg infusion in a similar humanized skin allograft BALB/c Rag2−/−cγ−/− model that at a Treg:PBMC ratio of 1:10 the immunosuppressive effect of Treg infusion was lost^27^. Using our humanized mouse allograft SCID/beige model similar observations were made (data not shown).

Here, we demonstrate that low numbers of Treg applied locally can successfully dampen skin inflammation. Where possible, this finding paves the way for the development of protocols that focus on local delivery, without the need for prior *ex vivo* Treg expansion.

## Results

### Intradermal injection of low dose freshly isolated Treg inhibits skin inflammation

**Figure 1:**
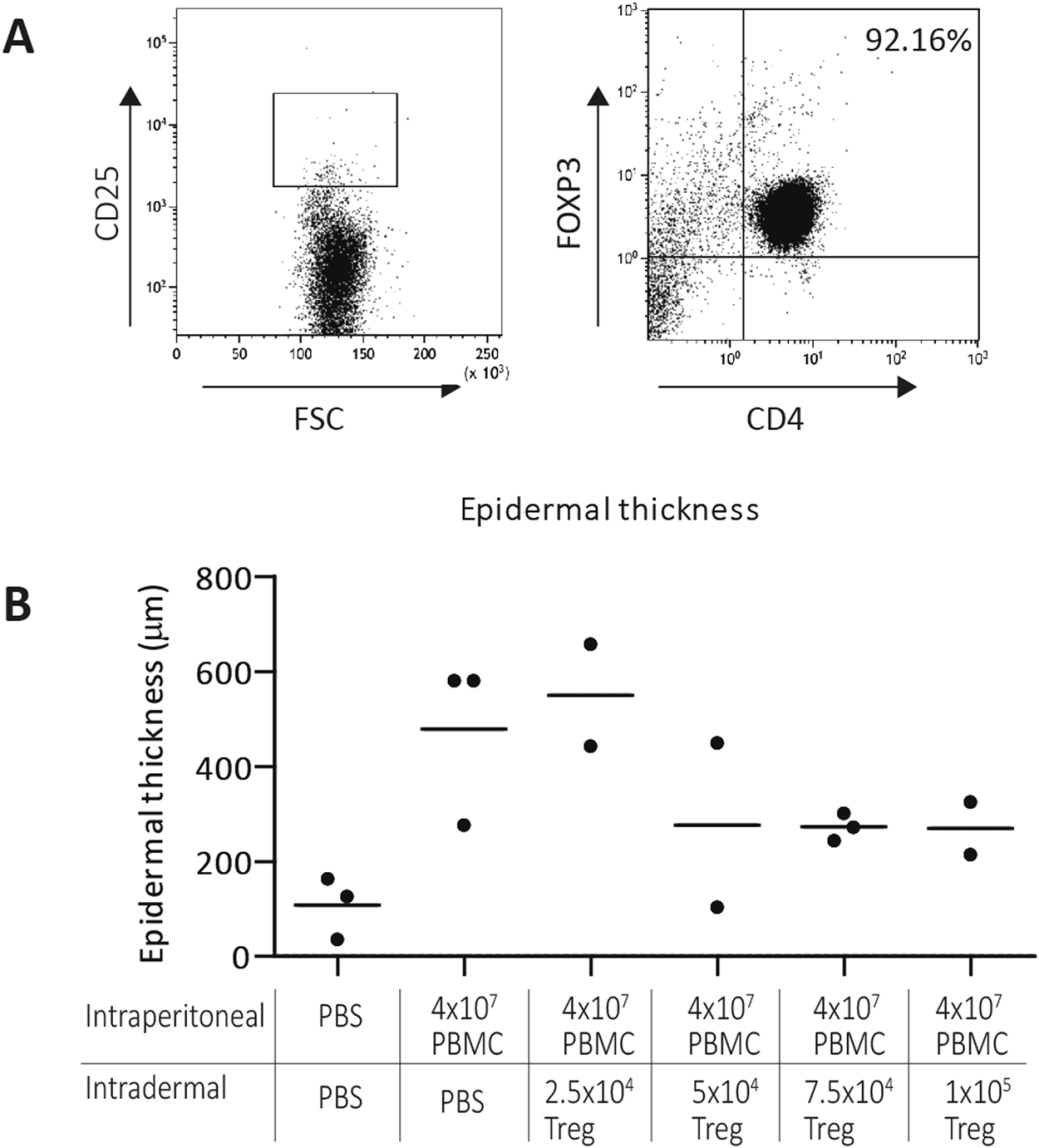
CD4+ CD25high gating strategy and dose response curve of freshly isolated non-expanded Treg. (**A**) Gating strategy for sorting CD4+ CD25high Treg from isolated CD4+ T cells, and flow cytometric analysis of FOXP3 expression after sorting. (**B**) Dose-response curve showing epidermal thickness of human transplanted skin in the huPBL-SCID-huSkin allograft model upon IP PBMC injection without or with intradermal injection of increasing doses of Treg (X-axis). Every dot in the figure represents data from a single mouse.

To assess the effectiveness of intradermally applied Treg in the inhibition of human skin inflammation, we first determined the minimal amount of intradermally injected Treg that were needed to inhibit epidermal thickening as part of the inflammatory process in the huPBL-SCID-huSkin allograft model. A range of 2.5–10 × 10^4^ high purity FACS sorted human Treg were injected intradermally together with 4 × 10^7^ allogeneic IP injected PBMC. FACS sorted Treg were typically >96% pure CD4^+^CD25^hi^ cells and more than 90% of these cells were FOXP3+ (Fig. 1A**)**. IP injection of human allogeneic PBMC alone induced epidermal thickening of the grafted human skin (PBS 126.0 µm (36–163 µm), PBMC 580.0 µm (276–580 µm), p = 0.0765), as also described previously^21^. We found that at least 7.5–10 × 10^4^ Treg were needed to inhibit this process (thickness: 272 µm (244–301 µm) and 269,5 µm (214–325 µm), respectively (Fig. 1B**)**).

**Figure 2:**
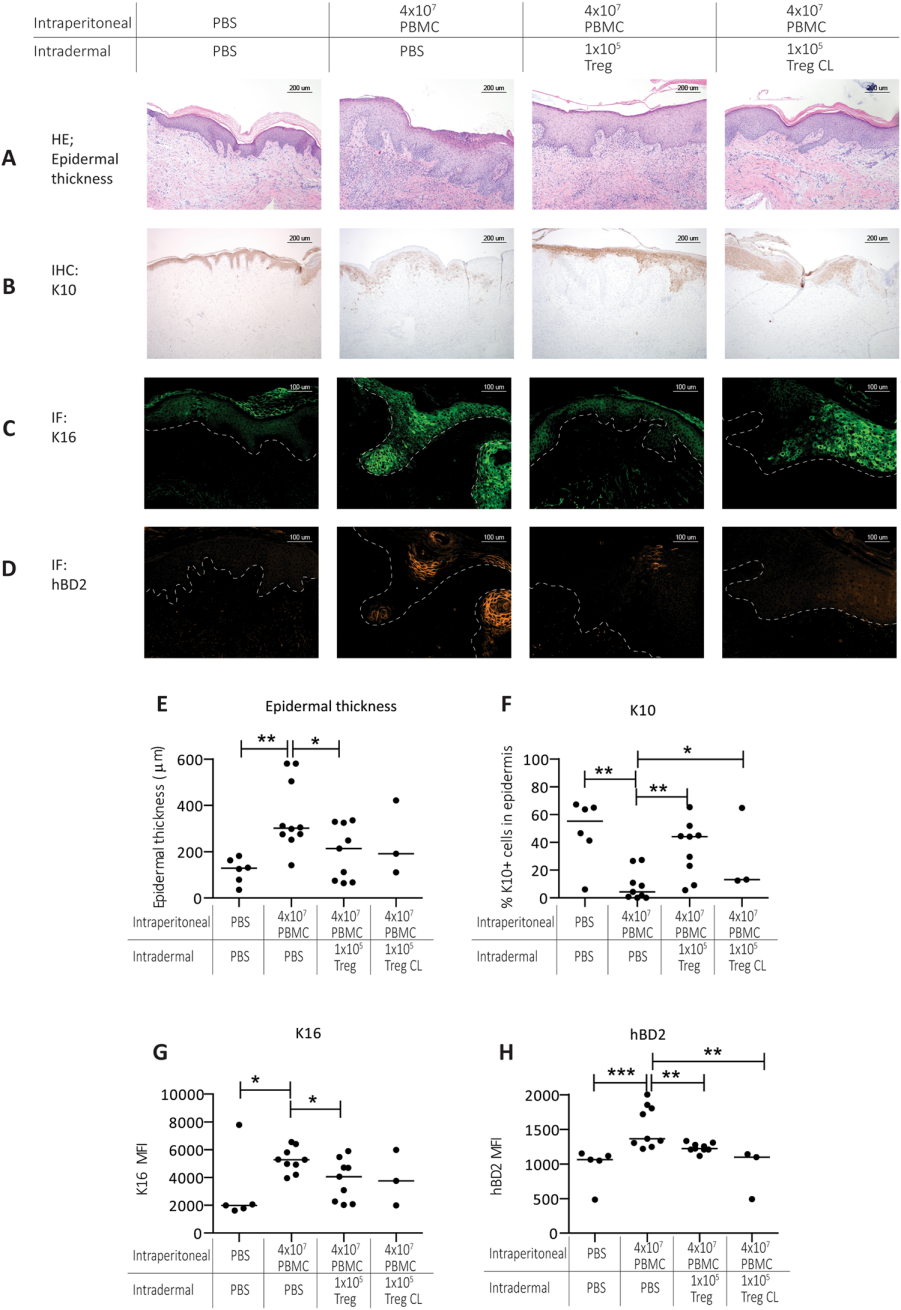
Inhibition of epidermal thickening, sustained K10 expression and absence of K16 and hBD2 up regulation in the epidermis of the human skin after intradermal Treg injection. Representative histology of human skin grafts from SCID beige mice 21 days after IP injection with huPBMC with or without intradermal low dose Treg injection showing: (**A**) epidermal thickness (HE staining), and the expression of (**B**). K10 (IHC), (**C**). K16 and (**D**). hBD2 (IF). The cell injection conditions are indicated at the top. The most right panel shows the effect of injecting Treg at the contralateral side of the transplanted skin (**E**–**H**). Cumulative data showing the median of the epidermal thickness, percentage K10, and median expression levels (MFI) of K16 and hBD2. Every dot in the figure represents a single mouse experiment (n = 3–10). Statistical significance was analyzed by the Mann Whitney U test. ^*^P < 0.05. ^**^P < 0.01.

Based on these results, we selected a dose of 1 × 10^5^ intradermally injected Treg and 4 × 10^7^ IP injected PBMC (a Treg:PBMC ratio of 1:400) to be used for subsequent experiments. Intradermal injection of this low dose of Treg prevented hyperkeratosis (Fig. 2A,E) (PBS 163.0 µm (36–212 µm), PBMC 301.3 µm (142–580 µm), PBMC + Treg 214.0 um (64–335.8 µm) (PBMC vs PBMC + Treg p = 0.0395), and prevented parakeratosis and acantosis, as well as the inflammation induced reduction of K10 expression (Fig. 2B,F) (PBS = 50.9% (24.6–67.2%), PBMC = 4.4% (0–27.3%), PBMC + Treg = 44.2% (5.6–65.2%) (PBMC vs PBMC + Treg p = 0.002)). Expression of K16 en hBD2 was analysed by immunofluorescence, and quantified by measuring the median fluorescent expression levels (MFI) in the epidermis (Fig. 2C,D). Low dose Treg injection inhibited the inflammation induced expression (MFI) of K16 (Fig. 2C,G) (PBS = 1883 (1609–2051), PBMC = 5267 (3950–6543) and PBMC + Treg = 4056 (2021–6993), PBS vs PBMC p = 0.0014; PBMC vs PBMC + Treg p = 0.0252) and hBD2 (Fig. 2D,H**)** (PBS = 1089 (1049–1153), PBMC = 1364 (1220–2005) and PBMC + Treg = 1221 (1116–1333), PBS vs PBMC p = 0.0014, PBMC vs PBMC + Treg p = 0.0039).

**Figure 3:**
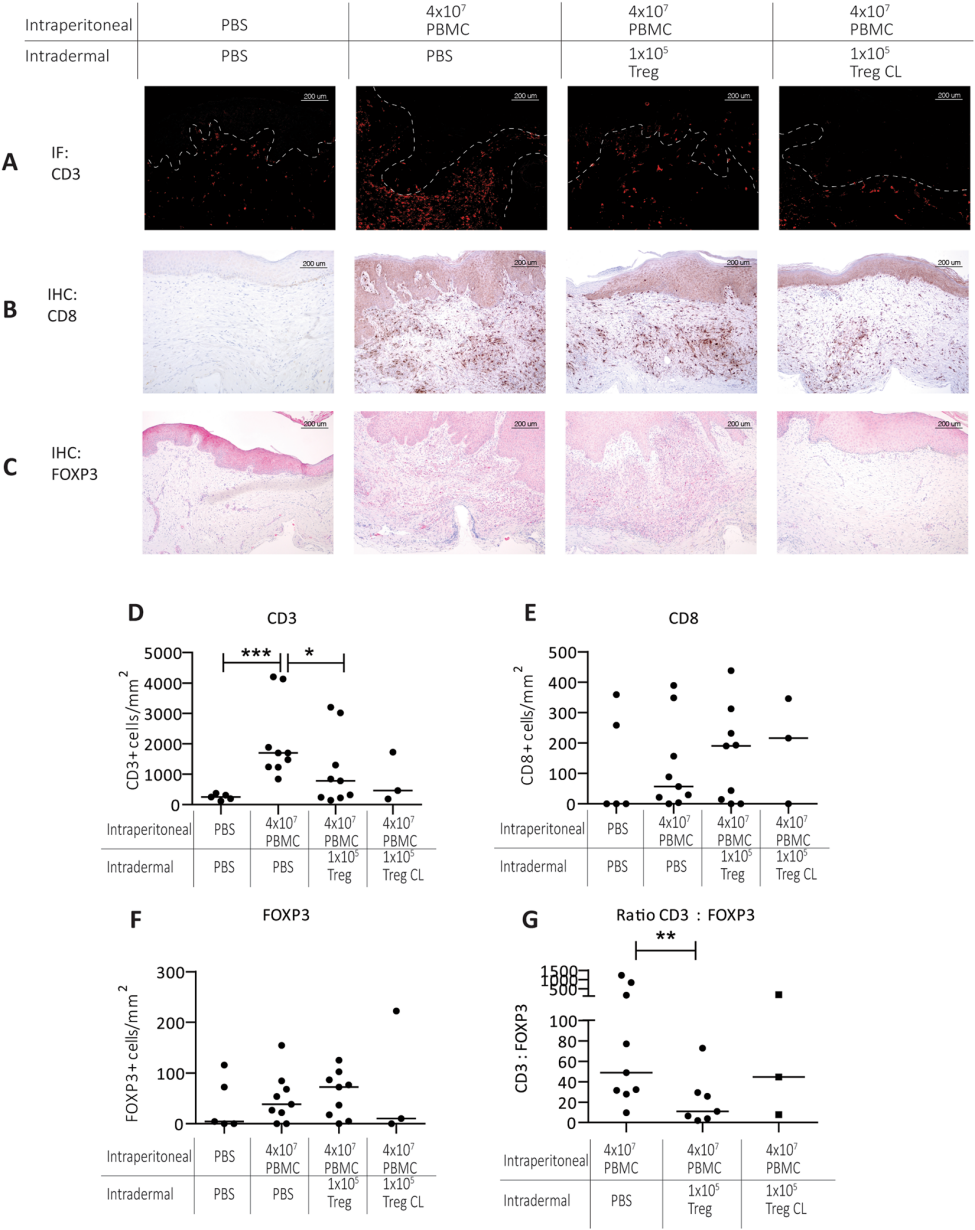
Intradermal Treg injection inhibits the T cell influx and promotes FOXP3+ cells in the human dermis. Representative histology of human skin grafts from SCID beige mice 21 days after IP injection with huPBMC with or without intradermal low dose Treg injection showing (**A**). CD3, (**B**). CD8 and (**C)**. FOXP3 cell counts per mm^2^ in the dermis (**D**–**F)**. Cumulative data figures showing the median of the cells counts. (**G**) Ratio of CD3:FOXP3 in the dermis. Every dot in the figure represents a single mouse experiment (n = 3–10). Statistical significance was analyzed by the Mann Whitney U test. ^*^P < 0.05. ^**^P < 0.01.

Skin inflammation in the huPBL-SCID-huSkin allograft model is largely driven by the influx of human T cells into the dermis^21^. Intradermal injection of low dose Treg significantly inhibited the influx of human CD3+ T-cells in the dermis (PBS = 282 (117–373) cells/mm^2^, PBMC = 1700 (842–4197) cells/mm^2^ and PBMC + Treg = 784 (142–3202) cells/mm^2^, PBS vs PBMC p = 0.0014, PBMC vs PBMC + Treg, p = 0.0252) (Fig. 3A,D). We observed that the injection of Treg did not influence the influx of CD8 + T cells (Fig. 3B,E), (PBS 0–359.3 cells/mm^2^ (median 0 cells/mm^2^), PBMC 0–389.5 (median 56.69/mm^2^), PBMC + Treg 0–437.8 cells/mm^2^ (median 190.6 cells/mm^2^); hence the observed reduction in CD3+ cells is likely due to a reduced influx of CD4+ T cells. Although we did not observe a significant increase in the absolute number of human FOXP3 + Treg in the human dermis after injection of Treg (Fig. 3C,F) (PBMC 0–154.7 cells/mm^2^, (median 38.6 cells/mm^2^), PBMC + Treg 0–125.1 cells/mm^2^, (median 72.1 cells/mm^2^)), a significant increase was found in the ratio of CD3:FOXP3(Fig. 3G) (PBMC median ratio 49:1, PBMC + Treg median ratio 11:1, p = 0.0082). This suggests that intradermal injection of Treg in the mouse skin leads to a relative increase in FOXP3+ cells in the human dermis and local inhibition of the inflammatory response.

Having established that intradermal injection of Treg in close proximity to the inflamed skin is effective, we next wondered if this close proximity is actually required to mediate the inhibitory effect of the Treg. Thus we set out to inject Treg at the contralateral (CL) side of the skin graft. Data are presented as the right column of Figs 2 and 3. The data show that also contralateral injection results in reduced epidermal thickening (epithelial thickness 111.2–421.4 µm (median 191 µm) p = 0.0182), restoration of the K10/K16 conversion (K10 = 12.5–64.8% (median 13.10), p = 0.05 and K16 MFI = 1985–3749 (median 2867) p = 0.0182, down regulation of hBD2 (MFI = 1098–1142 (median 1120), p = 0.0182), and reduced cell influx (CD3 = 185.5–460.6 cells/mm^2^ (median 323.1 cells/mm^2^), p = 0.0182.

### Low dose intradermal injection of Treg affects systemic repopulation of human lymphocytes

**Figure 4:**
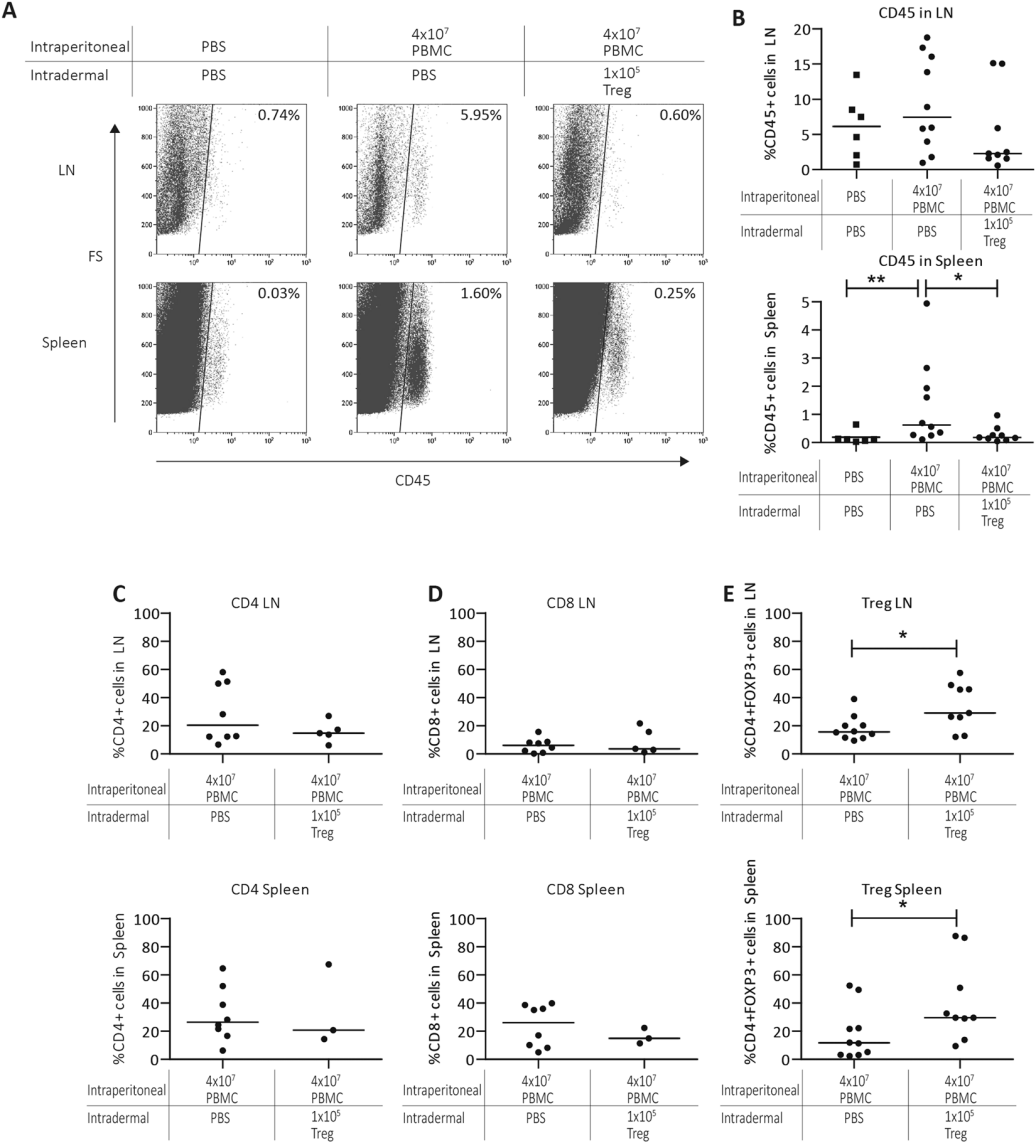
Intradermal Treg injection prevents repopulation of huPBMC and results in a relative increase of CD4+ FOXP3+ cells in the secondary lymphoid organs. (**A**) Representative flow cytometry pictures showing human CD45 expression in single cell suspensions from draining LN and spleen from SCID beige mice 21 days after IP injection with huPBMC with or without intradermal low dose Treg injection. Cumulative data figures showing the median percentages of human (**B**). CD45+ cells and (**C**). CD4+, (**D**). CD8+, (**E**) CD4+ FOXP3+ cells within the CD45+ cells in draining in LN (upper row) and spleen lower row). N = 3–10 Statistical significance was analyzed by the Mann Whitney U test. ^*^P < 0.05.

To assess whether next to local effects intradermal injection of Treg also exerted systemic effects we analyzed secondary lymphoid organs. After harvesting and processing spleen and draining lymph nodes (LN), the obtained cells were analyzed by flow cytometry. Following intraperitoneal injection of human-PBMC in the skin-transplanted immunodeficient SCIDbeige mice there is clear expansion of the infused T cells that is considered as repopulation, which can be visualized measuring the expression the human leukocyte marker anti-human-CD45 by flow cytometry^21,28^. IP injection of 4 × 10^7^ PBMC resulted in systemic repopulation of human CD45^+^ lymphocytes in the mouse spleen and LN, which is in line with previous findings^15,21^. We observed an average of 7.4% human CD45^+^ cells in the LN and 0.63% CD45^+^ cells in the spleen. Intradermal injection of 1 × 10^5^ Treg inhibits human lymphocyte repopulation in LN (2,28%, NS.) and spleen (0.2%, p = 0.0111). (Fig. 4A,B). Although the percentages of human CD4^+^ and CD8^+^ T cells within the CD45^+^ population were similar in LN and spleen (Fig. 4C,D), the percentage of CD45^+^CD4^+^FOXP3^+^ T cells were significantly increased in LN and spleen following intradermal Treg injection. (LN 15.6% in PBMC, 29.0% in PBMC + Treg p = 0.0175; Spleen 11.7% in PBMC, 29.6% in PBMC + Treg p = 0.0175) (Fig. 4E). Taken together, these results show that low dose intradermal Treg injection influences not only the local, but also systemic inflammatory response in the huPBL-SCID-huSkin allograft model.

## Discussion

Severe inflammatory skin diseases like severe psoriasis are often treated with biologics, which target the proinflammatory cytokine signaling pathways of TNFα, IL-23 and IL-17. Although these biologics are very successful in the treatment of psoriasis, a substantial group of patients does not respond to this treatment. This urges the quest for novel treatment modalities. Treg-based immune therapy might be a future option. Recently, successful clinical trials with Treg have been conducted in stem cell and solid organ transplantation as well as in (auto)inflammatory diseases such as type-1 diabetes and uveitis^9,12,29–32^. Also Treg based therapy is considered in the treatment of refractory Crohn’s disease^11^. An advantage of Treg therapy might be realization of infectious immune suppression, known as a self-perpetuating mechanism of immune suppression. Biologics on the other hand need to be administered life-long.

The application of Treg based therapy is studied in clinical transplantation and auto-inflammatory disease^2,4^, typically using intravenous infusion of large numbers of Treg (e.g. 1–10 million cells/kg in the ONE study^33^). To obtain high numbers of Treg these cells have to be expanded *ex vivo* prior to use^13,15–18^. *Ex vivo* Treg expansion is a critical and laborious step that also introduces the risk of Treg conversion into pro-inflammatory cytokine producing cells^19^. We here hypothesized that targeted delivery of Treg at the site of inflammation might require lower numbers of Treg, thereby circumventing large scale *ex vivo* expansion with its drawbacks. In the present study, we demonstrate that intradermal injection of very low numbers of freshly isolated, non-expanded, human Treg can inhibit skin inflammation in the humanized mouse huPBL-SCID-huSkin allograft model.

Systemic administration of Treg, either by intravenous or intra peritoneal injection, may well require high numbers of Treg because the injected cells will be distributed throughout the circulation and tissues and as a consequence a substantial part of the infused cells will be lost^34^. This appears not to be the case when Treg are injected locally, as we demonstrate here. As for now, we can only speculate why intradermal Treg injection is so efficient. The skin is a complex organ, that next to its strong barrier function, has an important role in the immune defence^35^ and reveals active trafficking of immune cells such as antigen presenting cells and T cells^36,37^. The local skin environment facilitates optimal migration to the draining lymphoid organs and control of the immune response. This is supported by skin based vaccination strategies that in humans resulted in highly efficient immunization^38^. Interesting in this respect are intradermal dendritic cell (DC) vaccination trials that resulted in superior antitumor T-cell induction and faster transit to draining LN^39–42^. Since intradermal Treg injection in our model led to inhibition of systemic human immune cell reconstitution, it is likely that intradermally injected Treg travel towards the draining LN and regulate the immune response at the level of the secondary lymphoid organs. The notion of a more systemic immune suppressive effect is further strengthened by our observation that injection of Treg at the contralateral side of the animal, and thus far distant from the human transplant, also inhibited inflammation of the graft. Although local intradermal injection of low Treg numbers seems to impair the systemic immune response, we advise scientist interested in the suppressing systemic inflammation to validate these findings.

Although our current work shows for the first time that small numbers of intradermally injected Treg inhibit inflammation *in vivo*, it appeared technically challenging to demonstrate where and how these little numbers of Treg exerted *in vivo* suppression. In our current work we focused on biodistribution analysis by both fluorescence and radioactive tracking of the injected Treg. CFSE-based cell labeling is possibly of interest for short term-tracking (ie 1–3 days), but appeared not suited to for long-term (3 weeks) biodistribution analysis of intradermal injected Treg (data not shown). Additionally we focused on radioactivity-based biodistribution analysis and survival of the injected Treg by radioactive 111Indium labeling of the cells combined with whole body scan SPECT-CT imaging. However, after multiple endeavors of labeling of human Treg with 111Indium-oxinate the labeling procedure turned out to be challenging; the labeling efficiency of the isolated Treg was poor and their survival after the labeling was low and unfortunately did not allow for biodistribution and survival analysis. Future studies should focus on optimizing Treg labeling and *in vivo* tracking procedures in order to demonstrate the survival and biodistribution of the intradermally injected cells and to further demonstrate their mechanism of action’.

In our model, we have been infusing Treg in the skin with the idea to prevent skin inflammation. The skin is an easily accessible organ, the question is if local delivery of Treg is feasible in case of solid organ transplantation or autoimmune disease such as type-1 diabetes (T1D) or Crohn’s disease, which are obviously less accessible. Our data suggests that intradermal Treg infusion promotes Treg migration to the secondary lymphoid organs and controls the immune response there. In fact, in human LNs we have previously demonstrated that Treg are activated and that a substantial number of the Treg is proliferating^43^. This suggests that secondary lymphoid organs are a physiological platform to organize Treg output. In case of Treg-based therapy in organ transplantation, T1D or Crohn’s disease intra nodal injection of Treg in the draining LNs might facilitate local control of the immune response. Intra nodal injection of dendritic cells is already successfully clinically applied in tumor vaccination protocols^44^.

Taken together, targeted intradermal administration of low dose Treg seems to offer a feasible approach for Treg therapy that omits the need for laborious and costly *ex vivo* Treg expansion and reduces the risk of Treg plasticity.

## Materials and Methods

### Humanized mouse model; huPBL-SCID-huSkin allograft model

The huPBL-SCID-huSkin allograft model used in this study is described in detail by de Oliveira *et al*.^21^. Female B17.B6-Prkdc^scid^Lyst^bg/Crl^ (SCID beige) mice, 8–12 weeks old (Charles River Breeding Laboratories) were transplanted with human skin from healthy individuals obtained after abdominal plastic surgery at Bauland kliniek (Mill, the Netherlands). After healing of the human skin (21 days), 4 × 10^7^ PBMCs were injected IP in the absence or presence of 2.5 × 10^4^–1 × 10^5^ intradermally injected Treg. All animal experimental procedures were in accordance with the international welfare guidelines and approved by the institutional animal ethical committee of the Radboud University in Nijmegen (DEC 2013–023). The use of human skin and peripheral blood were approved and in accordance with the regulations set by the Medical Ethical Committee for human research of the Radboudumc. Human skin and buffy coats (Sanquin Blood Bank, Nijmegen, the Netherlands) were obtained from healthy donors, who gave written consent for scientific use according to the declaration of Helsinki. All experiments were performed in accordance with relevant guidelines and regulations.

Mice were sacrificed 3 weeks after cell injection by orbita extraction followed by cervical dislocation. Terminating the experiment after 3 weeks allows us to study skin inflammation before skin is rejected. In rare cases rejection with GVHD like symptoms, like excessive proliferation of PBMCs in LN and/or spleen, are seen. These mice are excluded from the analysis after ending the experiment with the following exclusion criteria: > 5% CD45+ cells in spleen and/or >20% CD45+ cells in LN as measured with FACS.

### Regulatory T cell injection

PBMCs where isolated from buffy coats using ficoll density gradient isolation (Lymphoprep, Nycomed-Pharma AS, Oslo, Norway). To isolate human CD4+ CD25hi Treg, first CD4+ T cells were enriched using the RosetteSepTM (StemCell™ Technologies, Vancouver, Canada) human CD4^+^ T cell enrichment cocktail according to the manufacturers description, followed by high purity CD4^+^CD25^high^ cell sorting using a BD FACSAria cell sorter (BD Biosciences, Erembodegem, Belgium). For this purpose, cells were labeled with CD4-BV510 and CD25-Pe-Cy7(M-A251; BD Biosciences, New Jersey, USA). This typically resulted in >96% pure CD4^+^CD25^hi^ cells and more than 90% of these cells were FOXP3^+^. Without *ex vivo* expansion, high purity sorted Treg (or PBS, vehicle control) were injected intradermally in the mouse skin at 4 spots closely around the human skin graft, in a total volume of 100 µl PBS.

### Histology

Human skin grafts were fixed in neutral buffered 4% formalin (Mallinckrodt Baker Inc, Deventer, the Netherlands) for 4 hours and processed in a Tissue-Tek VIP tissue processor and embedded in paraffin. 6 µm sections of human skin transplants were stained with hematoxillin-eosin (HE) or processed for either immunohistochemical (IHC) or immunofluorescent (IF) staining. Keratinocyte differentiation was analyzed using antibodies against keratin 10 (K10, IHC, RKSE60, Sanbio, Uden, The Netherlands) and keratin 16 (K16, IF, LL025, BioTrend, Koln, Germany). Anti-Human β-defensin 2 (hBD2, IF, Ab9871, Abcam, Cambridge, UK) was used as a marker for the innate skin inflammatory response. For the detection of T cells the following antibodies were used: anti-CD3 (IF, A0452, DAKO, Glostrup, Denmark), anti-CD8 (IHC, 144B, Dako), and anti-FOXP3 (IHC, PCH101, eBioscience, ThermoFisher Scientific, Waltham, USA). For IHC, antibody stainings were visualized using the EnVision + system-HRP (Dako) combined with 3,39-diaminobenzidine tetrahydrochloride (DAB, brown metal enhanced DAB 1856090 Thermoscientific) or using the Labeled Streptavidin Biotin method (Universal LSAB Kit/AP; Dako) combined with Permanent Red (Dako). For IF, nuclei were stained and embedded using DAPI (fluormount-G with DAPI, eBioscience). The secondary antibody for K16 was AF488-conjugated donkey anti-mouse IgG (ThermoFisher); for hBD2 AF546-conjugated donkey anti-goat (ThermoFisher), and for CD3 AF647-conjugated donkey anti-rabbit (ThermoFisher). Sections were photographed using a microscope (AxioImager M2; Zeiss, Sliedrecht, the Netherlands) and a high resolution color camera for bright field microscopy (AxioCam 105 color, Zeiss) and a high resolution b/w camera for multichannel fluorescence microscopy (AxioCam 503 Mono).

### Image analysis of histology

Mean epidermal thickness was calculated using AxioVision software version 4.8 (Zeiss) or ZEN blue edition version 2.3 (Zeiss) as epidermal area divided by epidermal surface length. For quantification of K10 positive cells, images were saved at 10x objective magnification. The total epidermal area and K10 positive area were measured using ImageJ in the region of interest (ROI) and displayed as % K10 positive epidermal area. To determine the number of CD8+ and FOXP3+ cells, representative pictures were made at 10 × objective magnification. To quantify the cell counts in the dermis, a representative ROI was drawn from the lowest epidermal papilla till 300 µm dept in the dermis. Cells in this region were counted and expressed as the number of cells per mm^2^. For quantification of K16 and hBD2 positive cells, images were made at 20x objective magnification. The median fluorescent intensity (MFI) in epidermis was determined using ZEN blue (Zeiss). CD3 + cells were counted in the dermis using ZEN blue and expressed as the number of cells per mm^2^.

### Flow Cytometry

Spleen and mesenteric lymph nodes (LN) were harvested and cells were obtained by mashing over a 40 µm filter. Single cell suspensions of spleen and LN were phenotypically analyzed using a Navios multi-color flow cytometer (Beckman-Coulter, Mijdrecht, the Netherlands). Human leukocytes were identified using an antibody directed against the common human surface leukocyte marker CD45 (anti-human-CD45-KO (J33, Beckman-Coulter). For cell-surface staining of human CD4 and CD8 T cells, the following antibodies were used: anti-CD4-PC5.5 (13B8.2, Beckman Coulter), and anti-CD8-APC-AF700 (B9.11, Beckman-Coulter). For intracellular staining with anti-FOXP3-e450 (PCH101, eBioscience), cells were fixed and permeabilized by fix-perm treatment (eBioscience) according to manufacturers instruction. Data were analyzed using Kaluza software version 1.5a (Beckman-Coulter) and gates were set based on single staining and FMOs (Supplemental Fig. 1)

### Data and Statistics

Statistical analysis was performed using GraphPad Prism software version 5.03 (Graphpad Software Inc., San Diego, US). The median of the data points is displayed unless mentioned otherwise. Groups were compared using Mann Whitney U test. Differences with a p-value of <0.05 were considered significant and are indicated with an asterisk (^*^). p < 0.01 is indicated as^**^.

## Electronic supplementary material

Supplementary Figure 1
