# Supplementary material for: Intradermal injection of low dose human regulatory T cells inhibits skin inflammation in a humanized mouse model
Source: Sci Rep. 2018 Jul 3;8:10044. doi: 10.1038/s41598-018-28346-5 (PMC6030170; doi:10.1038/s41598-018-28346-5)
Supplement: Supplementary file 1 — Supplementary Figure 1 [file 41598_2018_28346_MOESM1_ESM.pdf]

Intradermal injection of low dose human regulatory T cells inhibits skin inflammation in a humanized mouse model.

Sija Landman[1], Vivian L. de Oliveira[1], Piet E.J. van Erp[2], Esther Fasse[1], Stijn C.G. Bauland[3] Irma Joosten[1] Hans J.P.M Koenen[1*]

[1] Radboud university medical center, department of Laboratory Medicine-Medical Immunology, Nijmegen, the Netherlands
[2] Radboud university medical center, department of Dermatology, Nijmegen, The Netherlands
[3] Bauland kliniek, Mill, the Netherlands

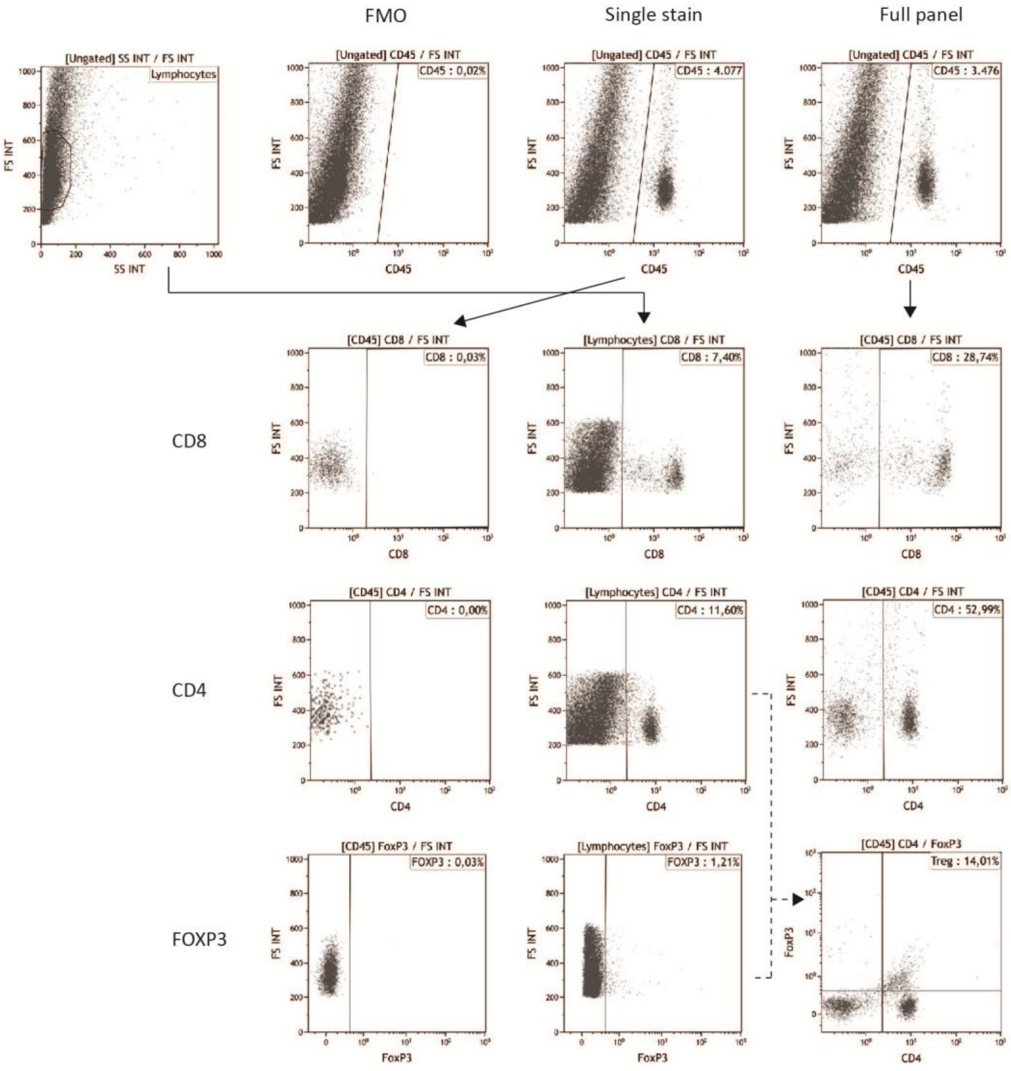

Supplementary figure 1. Gating strategy and marker settings for the expression of CD45, CD4, CD8 and FOXP3 on cells isolated from secondary lymphoid organs. An example showing data obtained using a mix of pooled human PBMCs and splenocytes from a non-transplanted, non PBMC-injected mouse.
